# Interprofessional education as a potential foundation for future team-based prevention of alcohol use disorder

**DOI:** 10.1186/s12909-023-04100-y

**Published:** 2023-02-21

**Authors:** Scott Edwards, Tekeda F. Ferguson, Sonia Gasparini, Donald E. Mercante, Patricia E. Molina, Tina P. Gunaldo

**Affiliations:** 1 Department of Physiology, School of Medicine, New Orleans, USA; 2 Comprehensive Alcohol-HIV/AIDS Research Center, New Orleans, USA; 3 Center for Interprofessional Education and Collaborative Practice, New Orleans, USA; 4 School of Public Health, New Orleans, USA; 5 Neuroscience Center of Excellence, School of Medicine, New Orleans, USA; 6 Louisiana State University Health Sciences Center at New Orleans, New Orleans, USA; 7 Department of Physiology Comprehensive Alcohol-HIV/AIDS Research Center, LSU Health Sciences Center-New Orleans, 1901 Perdido St. MEB 7205, 70112 New Orleans, LA USA

**Keywords:** Alcohol, Interprofessional education, Medical students, Dental students, Nursing students

## Abstract

**Background:**

Effective screening of alcohol use and prevention of alcohol use disorder (AUD) requires the continuous preparation of educated and confident providers across all health professions who will ideally work in close collaboration in their future practices. As one mechanism for achieving this goal, the development and provision of interprofessional education (IPE) training modules for health care students may cultivate beneficial interactions among future health providers early in their formative education.

**Methods:**

In the present study, we assessed attitudes about alcohol and confidence in screening and AUD prevention in 459 students at our health sciences center. Students represented ten different health professions (audiology, cardiovascular sonography, dental hygiene, dentistry, medicine, nursing, physical therapy, public health, respiratory therapy, and speech language pathology programs). For purposes of this exercise, students were divided into small, professionally diverse teams. Responses to ten survey questions (Likert scale) were collected via a web-based platform. These assessments were collected before and after a case-based exercise that provided information to students on the risks of excessive alcohol use as well as the effective screening and team-based management of individuals susceptible to AUD.

**Results:**

Wilcoxon signed-rank analyses revealed that the exercise led to significant decreases in stigma toward individuals engaging in at-risk alcohol use. We also discovered significant increases in self-reported knowledge and confidence in personal qualifications needed to initiate brief interventions to reduce alcohol use. Focused analyses of students from individual health programs uncovered unique improvements according to question theme and health profession.

**Conclusion:**

Our findings demonstrate the utility and effectiveness of single, focused IPE-based exercises to impact personal attitudes and confidence in young health professions learners. While additional longitudinal cohort follow-up studies are needed, these results may translate into more effective and collaborative AUD treatment in future clinical settings.

**Supplementary Information:**

The online version contains supplementary material available at 10.1186/s12909-023-04100-y.

## Introduction

The current study examined the ability of a brief interprofessional education (IPE) experience to influence the confidence and attitudes of health professional students regarding alcohol use and alcohol use disorder (AUD) prevention. IPE is defined as “when students from two or more professions learn about, from and with each other to enable effective collaboration and improve health outcomes” [1].

Alcohol is a leading contributor to global disease burden [2] based on its pathophysiological interactions with a number of organ systems and co-morbid disease processes [3, 4]. While most individuals control their alcohol drinking, an AUD can develop in vulnerable individuals and is defined by a gradual loss of control over drinking, the emergence of negative emotional symptoms over time, and a profound disruption of work and social life [5]. AUD affects approximately 10% of the population in the United States at any given time, and up to one-third of adults in the United States will meet criteria for AUD over their lifetime according to criteria from the Diagnostic and Statistical Manual of Psychiatric Disorders [6]. AUD represents a life-long psychiatric disease based on the persistence of craving symptoms and high propensity for relapse during attempted abstinence [7]. As such, it must be regularly screened for and treated as a complex health condition and threat to public health. While AUD represents a devastating and costly condition, the vast majority of individuals with AUD who could benefit from treatment do not receive it [8]. Because patients that suffer from AUD or are at risk of developing an AUD will encounter several health care professionals at regular intervals throughout their lifetime [9], effectively combating AUD will require the training and active involvement of a variety of health care providers. Health sciences centers may represent a critical nexus for AUD education, and this process may be greatly facilitated by incorporating IPE principles into curricular designs [10].

To limit the development or progression of AUD, effective screening, brief intervention, and referral to treatment (SBIRT) methods represent evidence-based and cost-effective strategies to reduce alcohol-associated morbidity and mortality [11–13]. The United States Preventive Services Task Force recommends screening for unhealthy alcohol use in primary care settings in adults 18 years or older, and providing persons engaged in risky or hazardous drinking with brief behavioral counseling interventions to reduce unhealthy alcohol use [14]. The use of SBIRT in patients exhibiting at-risk drinking has been shown to be highly effective, with a 43% reduction in heavy drinking after six months in one study [15]. Unfortunately, these practices are drastically under-utilized [16], making their ultimate efficacy hard to determine [17]. This also emphasizes the need to educate a variety of health professionals on the characteristics, challenges, and efficacy of SBIRT strategies [18].

Evidence suggests that provider attitudes are another important determinant of their ability to adopt effective education and training programs for AUD management [19]. Many health providers harbor negative and/or pessimistic attitudes toward patients suffering from AUD [20, 21]. Even with the right attitude, primary care physicians report several barriers to implementing SBIRT for substance use disorder treatment, including time constraints and a lack of training [22]. As another possible roadblock for SBIRT implementation and efficacy, healthcare providers often cite a lack of confidence in their ability to counsel patients on lifestyle issues such as responsible alcohol use [23]. Indeed, at-risk drinkers are rarely asked about their drinking trends [24] despite encountering a multitude of healthcare professionals throughout their lifetime [9].

Recent studies indicate that interprofessional healthcare teams are key for the successful and widespread implementation of SBIRT [25]. AUD education differs across academic programs based upon accreditation standards, faculty expertise and/or professional scope of practice. The diversity in education provides an opportunity for an interprofessional learning experience. Pre-professional students can learn more about AUD and their respective professional role, share their personal and professional perspectives about AUD, and discuss opportunities to address AUD as an interprofessional team. This type of exposure can help to minimize negative attitudes surrounding alcohol use and increase the number of educated and confident providers [26].

Mitchell and colleagues [27] found an improvement in student attitudes toward working with patients who have alcohol problems after an IPE experience. The IPE experience included dental hygiene and nursing students. However, only dental hygiene student attitudes were measured. Students engaged in a didactic lecture session, four standardized patient simulations, followed by a debriefing session. Student attitudes were measured before and after the didactic session and post-simulation. A significant positive change was noted after the didactic session, but not after the simulation. The focus of the current study intended to build upon the literature through expansion of programs and the inclusion of alcohol-related information through case-based learning.

Louisiana State University Health Sciences Center at New Orleans (LSUHSC-NO) houses an Alcohol and Drug Abuse Center of Excellence and a centralized office focused on interprofessional education and collaborative practice. Individuals from both areas have collaborated on developing IPE experiences since 2014. In 2015, our institution established a centralized office, the Center for Interprofessional Education and Collaborative Practice (CIPECP), to support faculty training and student learning. The primary mission of the CIPECP is to increase interprofessional learning for students enrolled across all of six schools at LSUHSC-NO (Allied Health, Dentistry, Graduate Studies, Medicine, Nursing, and Public Health) by assisting research and teaching faculty in the development, implementation, and assessment of meaningful IPE training.

Beginning in 2017, the CIPECP engaged first- and second-year students in a two-year longitudinal IPE curriculum, known as Team Up™: Commit to Compassion, Communication and Collaboration. The second year of Team Up™ included cases, one of them focused on AUD. The purpose of the IPE case discussion was to increase student exposure to the attributes of binge drinking and AUD, communicate school-based coverage of AUD screening within their curricula, and discuss a team-based plan for AUD prevention. These discussions were also expected to minimize negative perceptions about AUD among the students. The overall objective of the study was to answer the research question “Can a single brief IPE session impact student perception about alcohol use and their role in AUD prevention?”

## Methods

Our program was designed to promote interprofessional interactions among a variety of future health professionals early within their formative education at our health sciences center. Students in diversified team groups were provided an opportunity to discuss a simulated AUD patient case and related training materials (see Supplementary Materials), with assessments of their knowledge and perceptions regarding alcohol and AUD prevention occurring before and after the training exercise. The Theory of Planned Behavior supports the use of perception-based surveys. Specific to this study, a change in attitudes about AUD could be expected to change future behavior. In this case, a more positive attitude surrounding AUD can support a health professional in addressing the condition, although this important long-term outcome was not tested in the current study.

### Participants

A total of 459 students representing 10 health professions at LSUHSC-NO took part in the two-hour 2019 IPE experience, being organized into small, professionally diverse IPE groups consisting of around 10 members each. Students were in their second year of study of their respective program, and no specific inclusion or exclusion criteria were used for enrollment. The distribution of student groups is indicated in Table 1. LSUHSC-NO faculty facilitators from clinically diverse backgrounds circulated among student groups and were available to answer questions about the exercise, as well as to provide encouragement and direction for teams that might be slow in engagement. This educational study was conducted with LSUHSC-NO IRB approval (IRB #10,106).

**Table 1:** Number of students represented within each health profession

Health Professional Program	Students
Audiology	**11**
Cardiovascular Sonography	**5**
Dental Hygiene	**30**
Dentistry	**59**
Medicine	**173**
Nursing	**91**
Physical Therapy	**31**
Public Health	**32**
Respiratory Therapy	**8**
Speech Language Pathology	**19**

### Program and study design

For the current study, we designed and conducted an IPE training module aimed at improving basic knowledge related to the diagnosis and treatment of AUD in students within the first two years of their training programs. This training module was development by an expert faculty committee representing all six schools at LSUHSC-NO as part of Team Up™. The objectives of the AUD training case were to: (1) recognize the prevalence and frequency of AUD in the general population, (2) learn to identify AUD-related behaviors and symptoms among patients, (3) recognize the most frequent comorbid conditions associated with AUD, and (4) develop the skills to establish plans for intervention and/or a team-approach to holistic management of disease. A simulated AUD patient case (see Supplementary Material) was discussed with the objective of creating an integrated care and resource referral plan, with students communicating unique competencies as well as constraints associated with their own field of training.

The training course also focused on the following four competencies established by the Interprofessional Education Collaborative: (1) Values and Ethics (VE5) - Work in cooperation with those who receive care, those who provide care, and others who contribute to or support the delivery of prevention and health services and programs, (2) Roles and Responsibilities (RR3) - Engage diverse professionals who complement one’s own professional expertise, as well as associated resources, to develop strategies to meet specific health and healthcare needs of patients and populations, (3) Teams and Teamwork (TT4) - Integrate the knowledge and experience of health and other professions to inform health and care decisions, while respecting patient and community values and priorities/preferences for care, and (4) Interprofessional Communication (CC6) - Use respectful language appropriate for a given difficult situation, crucial conversation, or interprofessional conflict [28].

### Data collection and analysis

Likert scale-based assessments [29] obtained via internet-based (Moodle) survey questionnaires were obtained from IPE team groups both before and after the AUD case-based exercise. Changes in responses across all health professions students (n = 459) and within each student group were analyzed by Wilcoxon matched-pairs signed-rank testing. The Wilcoxon sum of signed ranks (W) was calculated, and significant differences were indicated by p < 0.05.

## Results

Survey responses from students utilized a 5-point Likert scale with responses ranging from 1 (strongly disagree) to 5 (strongly agree). Survey responses to CIPECP-generated questions (Qs) across all health professions students (n = 459) are shown in Table 2 (including both raw scores pre- and post-training exercise as well as score changes that reflect post-pre differences). The 10 questions were related to knowledge (Q2, Q6) and perceptions (all other questions) associated with alcohol use and AUD prevention. Overall, we found significant score changes in association with 6 of the 10 questions presented. The largest changes occurred in Q7 (reflecting decreased stigma regarding individuals who might exhibit problematic drinking), Q6 (reflecting increased confidence expressed by the students to initiate brief interventions related to drinking), and Q2 (reflecting increased knowledge in students to advise patients about responsible drinking). Other significant improvements were found in Q8 (reflecting reduced embarrassment in the students in relation to engaging patients about their drinking), Q5 (reflecting increased student understanding of disease-based conceptualizations of AUD), and Q4 (reflecting an increased belief in students that all health professionals share a role in AUD intervention and treatment). We did not observe significant changes in four of ten questions presented (Q1, Q3, Q9, and Q10). These questions related to treatment timing (Q3 and Q9), belief in the role of the student’s particular profession in AUD interventions (Q1), and ethical appraisal of alcohol use (Q10).

**Table 2:** Changes in responses across students from all health professions (n = 459)

Survey Question	Pretest Mean (SD)	Posttest Mean (SD)	Mean Change	Sum of Signed Ranks (W)	P Value
Q1: I believe my own profession has a role to play in brief interventions when alcohol misuse is suspected in a patient.	**4.33 (0.79)**	**4.37 (0.78)**	**0.04**	**720**	**0.309**
Q2: I feel I have the appropriate knowledge to advise my patients about responsible drinking and the problems associated with alcohol misuse.	**3.98 (0.84)**	**4.15 (0.79)**	**0.17**	**5075**	< 0.001*
Q3: Health professionals who identify alcohol problems early can improve the chances of treatment success.	**4.47 (0.63)**	**4.52 (0.60)**	**0.05**	**1219**	**0.113**
Q4: All health professionals in the US share the responsibility of intervening when a patient is suspected of having an alcohol problem.	**4.28 (0.77)**	**4.36 (0.72)**	**0.08**	**2386**	0.014*
Q5: I believe alcohol problems are beyond the control of the person affected.	**3.04 (1.02)**	**2.93 (1.11)**	**-0.11**	**-3661**	0.010*
Q6: I believe that I have the personal qualities required to initiate brief interventions relating to responsible drinking.	**3.88 (0.78)**	**4.06 (0.77)**	**0.18**	**5428**	< 0.001*
Q7: In my private life I would avoid people whom I suspect to have problems with alcohol.	**2.73 (1.01)**	**2.53 (1.05)**	**-0.20**	**-5861**	< 0.001*
Q8: I would feel embarrassed asking patients about their use of alcohol.	**2.21 (0.94)**	**2.10 (0.95)**	**-0.12**	**-3028**	0.001*
Q9: People with an alcohol problem can only be effectively treated when they hit ‘rock bottom’.	**1.92 (0.96)**	**1.86 (0.94)**	**-0.06**	**-1384**	**0.128**
Q10: People should have the right to use alcohol as they wish within the confines of their own home.	**3.25 (0.99)**	**3.24 (1.01)**	**-0.01**	**-283**	**0.741**

Following the exercise, students collectively endorsed the notion that health care professionals as a whole are responsible for AUD-related interventions (Q4; p = 0.0064). However, when asked about their specific profession’s role in implementing brief interventions, no significant increases were found (Q1; p = 0.1671). This finding led to us to consider whether specific student groups endorsed other specific attitudes based on their individual professions. Table 3 shows significant changes in responses listed by specific health profession. It should be noted that statistical analyses of responses from certain under-represented professions were likely insufficiently powered to detect differences (e.g., Respiratory Therapy, which was represented by only eight students). With this caveat, we found several profession-specific changes in responses to specific questions. In each of these cases, the directionality of change for each question was identical between the specific health professional group and the combined student analysis from Table 2. Interestingly, nursing students displayed a significant change in Q1 response (reflecting a belief that their specific profession has a role in implementing brief AUD interventions), a sentiment apparently not shared by any other profession or even across all students collectively.

**Table 3:** Significant changes in responses within each student group

Student Group & Survey Question	Pretest Mean (SD)	Posttest Mean (SD)	Mean Change	Sum of Signed Ranks (W)	P Value
Dentistry (Q7)	**2.88 (0.95)**	**2.42 (0.97)**	**-0.46**	**-307**	< 0.001*
Dentistry (Q6)	**3.90 (0.78)**	**4.22 (0.65)**	**0.32**	**190**	0.003*
Medicine (Q5)	**2.99 (1.10)**	**2.76 (1.16)**	**-0.23**	**-956**	< 0.001*
Medicine (Q2)	**4.20 (0.82)**	**4.35 (0.71)**	**0.15**	**468**	0.002*
Medicine (Q7)	**2.72 (1.08)**	**2.56 (1.14)**	**-0.16**	**-477**	0.004*
Medicine (Q6)	**4.11 (0.70)**	**4.22 (0.71)**	**0.11**	**463**	0.011*
Nursing (Q2)	**4.02 (0.71)**	**4.29 (0.58)**	**0.26**	**242**	< 0.001*
Nursing (Q1)	**4.33 (0.84)**	**4.54 (0.56)**	**0.21**	**171**	0.015*
Nursing (Q7)	**2.71 (1.04)**	**2.56 (1.01)**	**-0.15**	**-182**	0.040*
Physical Therapy (Q2)	**3.48 (0.77)**	**3.87 (0.67)**	**0.39**	**69**	0.014*
Physical Therapy (Q6)	**3.48 (0.85)**	**3.81 (0.79)**	**0.33**	**66**	0.037*
Public Health (Q8)	**2.13 (0.94)**	**1.78 (0.79)**	**-0.35**	**-21**	0.031*
Public Health (Q6)	**3.66 (0.79)**	**3.97 (0.74)**	**0.31**	**85**	0.041*

## Discussion

Our AUD-based training exercise both informed early learners about the potential risks of alcohol use as well as provided guidelines and resources for the screening and treatment of AUD. Students were able to learn together as a foreshadowing of future interprofessional practice. This intervention produced significant decreases in stigma associated with alcohol use, highly relevant for potential AUD patients. Our work also discovered substantial improvements in student knowledge, confidence, and other personal qualities necessary for brief interventions aimed at limiting problematic alcohol use (Table 2). A more granular analysis of answers to each question from students representing individual health professions found distinct improvements according to school and question theme. Importantly, these findings could help inform the most effective timing of alcohol and substance use disorder education within these schools and programs (e.g., earlier or later in the curricula; Table 3). Altogether, our findings demonstrate the advantage of targeted IPE exercises to improve critical attitudes and future confidence to facilitate collaborative AUD treatment in interdisciplinary settings.

### Importance of impacting AUD treatment across multiple points of care

A recent qualitative analysis of barriers for screening practices interviewed a host of key interprofessional stakeholders (including patients, primary care providers, nurses, and medical assistants), and their findings emphasized a lack of training as a barrier, as well as the need for more universal screening practices [30]. Patients voiced concerns over the consequences of disclosing substance use, but despite this risk for stigma, also expressed that the primary care provider should play a central role in substance use screening and interventions. Indeed, patients generally support alcohol screening by their health care providers, and patients who are screened and counseled report having received higher-quality primary care overall [31, 32].

Less than 10% of patients discuss alcohol use with their personal physician [33]. Many health care providers are hesitant to ask patients about alcohol consumption due to the perception that screening may offend patients. However, a study of walk-in dental clinic patients found that over 75% of patients were in favor of dentist inquiries and advice regarding alcohol use [34]. Extending this finding, similar positive attitudes about alcohol screening were found in trauma inpatients [35] and in women receiving prenatal care [36]. In addition, an overwhelming majority (84%) of hospitalized patients responded favorably to SBIRT screening from nurses [37]. This would appear to be particularly important since our data indicate that nurses may uniquely adopt a primary responsibility for brief interventions for alcohol misuse (Table 3, **Q1**). However, our findings suggest that many other health professionals can also be effective in this regard. Indeed, impacting patients at such diverse points in time and location across their longitudinal care could lead to significant improvements in population-level health outcomes in relation to AUD.

### Connections to existing literature and limitations

The present study confirms and extends results from an assessment of a previous AUD-related IPE exercise at our institution that revealed significant student improvements in identifying the Alcohol Use Disorder Identification Test (AUDIT) as a useful screening tool, recognizing binge drinking patterns, and understanding the biomedical consequences of AUD [38]. More recently, Pervanas and colleagues [39] conducted a formal SBIRT training exercise in an IPE setting, after which over 70% of participants (including pharmacy, nursing, medicine, behavioral health, and physician assistant students) reported being confident in their ability to screen potential substance use disorder patients and refer them to treatment. A majority of students also considered the asynchronous online-based exercise to be extremely effective in facilitating interactions and increasing knowledge of the roles and responsibilities of other health professions. Such online formats may also better model how the bulk of interprofessional interactions may take place now and in the future. Our findings of widespread decreases in AUD-related stigma post-training are also in accordance with a recent study that found increased AUD-related knowledge along with decreased stigma in an interprofessional setting [40].

Two limitations of the current study are the absence of validated assessments and the relatively small sizes of certain student populations that may have precluded the discovery of effects in specific professions. For example, using the Alcohol and Alcohol Problems Perception Questionnaire, Mitchell and colleagues [27] described significant improvements in role security following an SBIRT-based IPE training exercise in a larger cohort of dental hygiene students (n = 67). Our single-site study would have also been improved by the inclusion of students from different institutions to enhance generalizability and better understand inter-institutional factors [41].

### Future directions

Several tailored customizations to the IPE training experience presented here can be envisioned. A critical extension would be the addition of scenario-based surveys and/or incorporation of screening demonstrations by professionals or the students themselves, which we are planning for future cohorts. Future exercises could also emphasize marginalized populations at heightened risk for AUD. For example, people living with HIV are less likely to receive evidence-based alcohol-related care [42]. Transcultural factors should be incorporated in terms of both inclusion of diverse student learners as well as in the sensitive provision of interprofessional AUD care to diverse populations [43]. Timing of IPE exercises within a curriculum may also be important. The current exercise was provided to second-year health professions students due in part to scheduling feasibility but also to target trainees in their more formative years of health care training. A recent study indicated that while students agreed that alcohol and substance misuse represented an important interprofessional topic for first-year students, they also noted the difficulty of addressing these cases early in their professional development [44]. Future work should attempt to extend these practices to more senior students and into areas of post-graduate education [26]. However, conducting SBIRT-related IPE at these levels will require an appreciation of the assumptions, divergent characteristics, and unique strengths of each profession including profession-specific competencies that may change over time [45]. Tetrault and colleagues [46] successfully designed and executed a multi-specialty graduate medical education curriculum on SBIRT for alcohol and substance use, including contributions from internal medicine, psychiatry, obstetrics and gynecology, emergency medicine, and pediatrics residents. While perhaps not considered part of the traditional health care team, the incorporation of social workers as SBIRT instructors in medical resident education has also proven very effective [47]. Another recent study utilizing an interprofessional simulation for nursing and social work students exposed the need for further training, particularly in crisis situations such as acute alcohol withdrawal seizure management when effective interprofessional communication might be compromised [48].

The combined psychological and pathophysiological aspects of AUD symptomatology should incentivize collaborative practice between behavioral, psychosocial, and primary care elements across all health professions [49]. With these objectives, targeted IPE training exercises such as those described here and being deployed across other health science centers [50, 51] could potentially provide a foundational basis for improving future AUD prevention and care. However, additional longitudinal cohort follow-up studies are needed to determine how interprofessional education translates into more effective practice.

## Electronic supplementary material

Below is the link to the electronic supplementary material.

Supplementary Material 1

## Data Availability

All data and materials available upon reasonable request from the corresponding author.
