# Supplementary material for: Interprofessional education as a potential foundation for future team-based prevention of alcohol use disorder
Source: BMC Med Educ. 2023 Feb 21;23:126. doi: 10.1186/s12909-023-04100-y (PMC9945599; doi:10.1186/s12909-023-04100-y)
Supplement: Supplementary file 1 — Supplementary Material 1 [file 12909_2023_4100_MOESM1_ESM.pdf]

TEAM UP

commit to

COMPASSION, COMMUNICATION, COLLABORATION

Alcohol Use Disorder

After the small-group discussion IPE session, students should be able to

- Work in cooperation with those who receive care, those who provide care, and others who contribute to or support the delivery of prevention and health services and programs – Values and Ethics (VE5).
- Engage diverse professionals who complement one's own professional expertise, as well as associated resources, to develop strategies to meet specific health and healthcare needs of patients and populations – Roles and Responsibilities (RR3).
- Integrate the knowledge and experience of health and other professions to inform health and care decisions, while respecting patient and community values and priorities/preferences for care – Teams and Teamwork (TT4).
- Use respectful language appropraite for a given difficult situation, crucial conversation, or interprofessional conflict - Interprofessional Communication (CC6).

Alcohol Use Disorder (AUD) is a serious public health problem. Approximately 16 million people in the U.S. have an AUD. An estimated 88,000 people die from alcohol-related causes annually, making alcohol the 4th leading preventable cause of death in the U.S. Once viewed as a moral failing or character flaw, AUD is now widely recognized as a chronic brain disease with potential for both recovery and recurrence.[1] Approximately 6.2 percent or 15.1 million adults in the United States ages 18 and older suffered from AUD in 2015. This includes 9.8 million men and 5.3 million women. Adolescents can be diagnosed with AUD as well, and in 2015, an estimated 623,000 adolescents ages 12–17 suffered from AUD.[2]

What is a Standard Drink?[3]

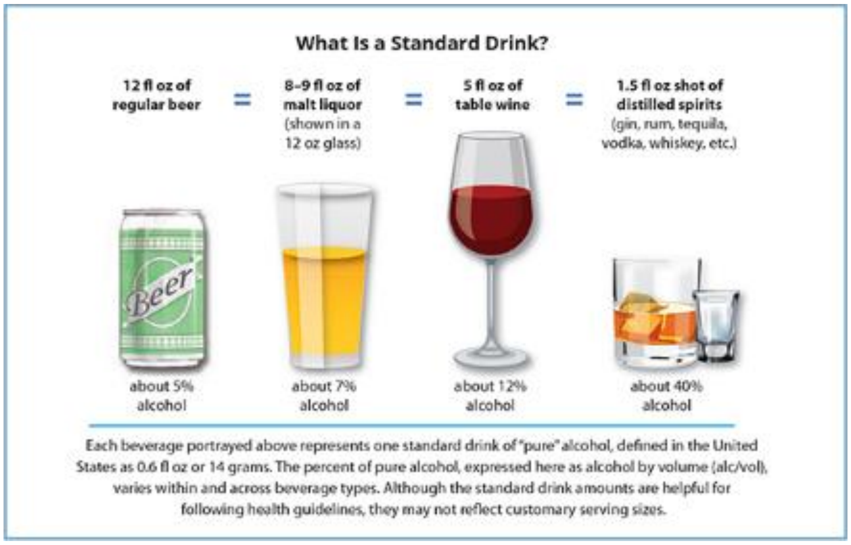

Q1. How do the standard drink sizes indicated above compare to what you are used to serving or receiving?

The Physiological Effects of Drinking at each Blood Alcohol Level[4]:

.02-.03	Slight euphoria & loss of shyness, mild relaxation. No loss of co-ordination.
.04-.06	Feeling of well-being, relaxation, lower inhibitions. Euphoria.
.07-.09	Continuing euphoria. Some impairment of balance, speech, vision, reaction time. Reduced judgement, caution & self control.
.10-.12	Loss of good judgement. Significant impairment of motor co-ordination. Slurred Speech. Euphoria.
.13-.15	Lack of physical control, gross motor impairment. Increasing dysphoria (anxiety & restlessness). Severly impaired judgement.
.16-.19	Dysphoria, confusion. Possible nausea.
.20	May need help to stand or walk. Possible loss of memory, nausea & vomiting.
.25	Severe impairment of mental, physical & sensory function.
.30	Stupor. Possible loss of consciousness.
.40	Onset of coma. Possible death due to respiratory arrest.

Binge Drinking[5]:

- The National Institute on Alcohol Abuse and Alcoholism (NIAAA) defines binge drinking as a pattern of drinking that brings blood alcohol levels to 0.08 g/dL. This typically occurs after 4 standard drinks for women and 5 drinks for men—in about 2 hours.
- The Substance Abuse and Mental Health Services Administration (SAMHSA) defines binge drinking as 5 or more alcoholic drinks for males or 4 or more alcoholic drinks for females on the same occasion (i.e., at the same time or within a couple of hours of each other) on at least 1 day in the past month.

Q2. Is the definition above representative of how you define binge drinking?

Heavy Alcohol Use[5]:

- SAMHSA defines heavy alcohol use as binge drinking on 5 or more days in the past month.

NIAAA's Definition of Drinking at Low Risk for Developing Alcohol Use Disorder (AUD)[5]:

- For women, low-risk drinking is defined as no more than 3 drinks on any single day and no more than 7 drinks per week.
- For men, it is defined as no more than 4 drinks on any single day and no more than 14 drinks per week.
- NIAAA research shows that only about 2 in 100 people who drink within these limits will develop an AUD.

Q3. Have a discussion on the importance of the team having the same knowledge of key terms related to AUD.

Q4. In your program, what do you learn about responsible drinking and Alcohol Use Disorder (AUD)?

Q5. In your program,

- **do you learn about AUD recognition?**
- **do you learn about AUD screening? What evidence-based screening tools are available and what questions are asked on the screening tools?**

John Boudreaux is a gay-closeted 40-year old white male who is married and has 2 children. John was taken to the University Medical Center (UMC) emergency room after a car accident. John was identified as the driver of the car. John presents to the emergency room with a blood alcohol level of .12, a chipped front tooth and neck pain.

Q6. HIV infection and emotional disorders (depression, anxiety) are sometimes co-morbid with AUD. Refer to John's responses on the intake form. In addition to the questions noted below, develop a list of diagnostic tests or questions that the team could ask John to determine if he may have an AUD or related co-morbidity?

Intake form:

**HABITS**		**COMMENTS**
Do you exercise?	☑ Yes ☐ No	I run in my neighborhood or go to a local gym
How often do you exercise?		I run in my neighborhood or go to a local gym a few times a week
Do you always use a condom during sex?	☐ Yes ☑ No	Never, since wife is on oral contraception.
Do you chew/smoke tobacco (per day)?	☐ Yes ☑ No	
Do you drink alcohol?	☑ Yes ☐ No	
How much alcohol do you drink?		I share a bottle of wine with my wife over dinner. I sometimes drink with friends at the end of a stressful week at work.
Do you use intravenous drugs?	☐ Yes ☑ No	
Do you consume caffeine (per day)	☑ Yes ☐ No	I drink a cup of coffee in the morning.
Do you have trouble sleeping?	☑ Yes ☐ No	Sometimes
Describe your eating habits?		I follow a Mediterranean diet
Do you eat out more than twice a week?	☐ Yes ☑ No	
Does drinking ever have a negative social impact on your life?	☐ Yes ☑ No	
Do you wish you could drink less?	☑ Yes ☐ No	Sometimes
Have you been injured due to drinking?	☐ Yes ☑ No	
Have you fallen within the past month?	☐ Yes ☑ No	

Q7. AUD use can have multiple effects on the body and public health outcomes. What assessments/ laboratory tests or public policy would you recommend and why?

Q8. Discuss how your team can use the American Society of Addiction Medicine's placement criteria?

Q9. John is discharged from UMC emergency room with multiple follow-up orders and consultations. John lives in Independence, LA. What health professionals would you recommend to become a team member and why?

Q10. Provide a specific example of how your team, working in the same physical space, can address responsible alcohol drinking or possible AUD.

Q11. Provide a specific example of how your team, providing care in different physical locations, can address responsible alcohol drinking or possible AUD.

References:

1. NIH.gov
2. NIAAA.nih.gov
3. https://www.niaaa.nih.gov/alcohol-health/overview-alcohol-consumption/what-standard-drink
4. http://wnewnl.blogspot.com/2015/01/understanding-your-bac-can-help-you.html
5. https://www.niaaa.nih.gov/alcohol-health/overview-alcohol-consumption/moderate-binge-drinking
6. https://pubs.niaaa.nih.gov/publications/strategicplan/niaaastrategicplan.htm
